# Supplementary material for: Splice-Junction-Based Mapping of Alternative Isoforms in the Human Proteome
Source: Cell Rep. Author manuscript; Available in PMC 2020 Jan 15. (PMC6961840; doi:10.1016/j.celrep.2019.11.026)
Supplement: 3 [file NIHMS1546469-supplement-3.zip › DF2/PXD000561/Liver-52-Q96HR9-NVKPSQTPQPK.pdf]

A

Predicted sequence disorder and sequence features of Q96HR9

Peptide: NVKPSQTPQPK Junction: sp|Q96HR9|REEP6_HUMAN|ENSG00000115255|SE2|15417|chr19|1496453|1496671|+0|r523|T1 TrNovel: FALSE

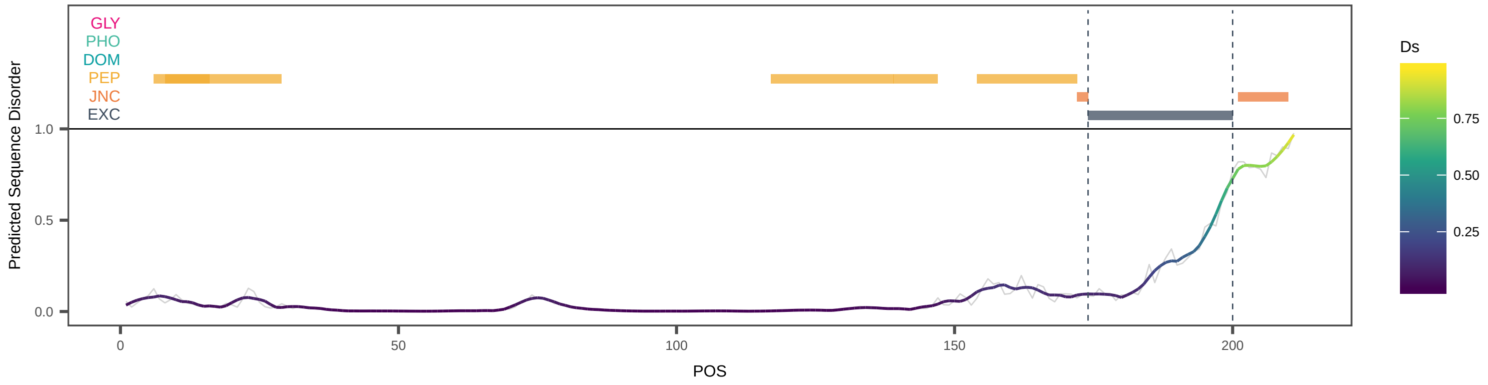

B

Distribution of sequence disorder in excised vs. mapped and non−excised regions of protein

M−W P−value vs. mapped: 7.01e−11 vs. non−excised: 5.67e−12

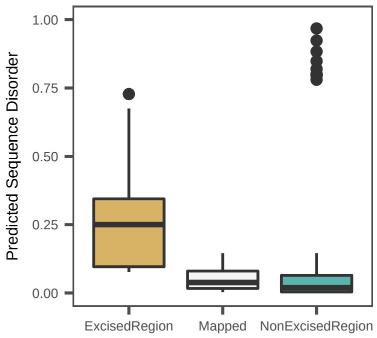

C
